# The organizational effects of oxytocin on the central expression of estrogen receptor α and oxytocin in adulthood

**DOI:** 10.1186/1471-2202-8-71

**Published:** 2007-09-07

**Authors:** Kristin M Kramer, Shigeto Yoshida, Eros Papademetriou, Bruce S Cushing

**Affiliations:** 1 Department of Biology, University of Memphis, Memphis, TN 38152, USA; 2 The Brain-Body Center, Department of Psychiatry, University of Illinois at Chicago, Chicago, IL 60612, USA; 3 Department of Biology, University of Akron, Akron, OH 44325, USA

## Abstract

**Background:**

Previous studies have demonstrated that neonatal manipulation of oxytocin (OT) has effects on the expression of estrogen receptor α (ERα) and the central production of oxytocin observed in juveniles (at weaning, 21 days of age). The goal of this study was to determine whether the effects of neonatal manipulation of OT last into adulthood, and if the effects differ from those observed during the early postnatal period. On the first day of life, prairie voles (*Microtus ochrogaster*) received one of three doses of OT (High, 3 μg; Med, 0.3 μg; Low, 0.03 μg), an OT antagonist, or isotonic saline. Another group was handled, but not injected. Then as adults, brains were collected, sectioned, and stained for ERα or OT using immunocytochemistry.

**Results:**

In females, treatment with OT increased the expression of ERα immunoreactivity in the ventral lateral septum (0.03 μg) and the ventromedial nucleus of the hypothalamus and central amygdala (0.3 μg). In males, OT antagonist increased ERα expression in the bed nucleus of the stria terminalis. There was no apparent effect of OT on the number of cells producing OT in the paraventricular nucleus of the hypothalamus.

**Conclusion:**

The current results suggest that neonatal manipulation of OT has long-term organizational effects on the expression of ERα in both males and females. The lack of effect on OT neurons in the paraventricular nucleus suggests that some developmental effects of OT previously observed in weanlings do not persist into adulthood. Developmental effects of OT on ERα patterns were sexually dimorphic, dose-dependent, and site-specific.

## Background

Recent studies indicate that during the neonatal period oxytocin (OT) has an organizational effect within the CNS. Neonatal manipulation of OT affects neuronal activity, as indicated by the expression of c-Fos in neonates [1], alters the number of OT neurons in the paraventricular nucleus of the hypothalamus (PVN) of weanlings [2], and the distribution of estrogen receptor alpha (ERα) in weanlings [3]. The effects of neonatal manipulation of OT are sexually dimorphic and site specific [1-3]. Given that neonatal manipulation of OT affects mechanisms that regulate physiological and behavioral responses it is not surprising that neonatal manipulations also affect a variety of behaviors, many of which occur in adults [4-7]. Neonatal manipulations of OT affect behaviors such as mating bout frequency [4], alloparental behavior [8], and behavioral reactivity to social isolation [9]. Aside from a recent report on developmental effects of OT on the pituitary in adults [10], the effects of early exposure to OT on the CNS have been characterized primarily during the early postnatal development period up to and including weaning [2,3]. Much remains to be known about the developmental effects of OT on the adult CNS. Therefore one of the goals of this study was to determine the effects of neonatal manipulation of OT on the production of OT in the PVN and the central distribution of ERα over a longer period of time. Previous studies of the effects on neonatal manipulation of OT have used a single dose of OT (3 μg), and while these studies have produced significant results the dose is pharmacological. Although no data are available on endogenous plasma OT levels in pups, data from adults suggest that a dose of 3 μg likely results in very high OT levels as it is 4 orders of magnitude higher than plasma concentrations in adult prairie voles [11]. Thus, a second objective was to determine whether developmental effects are also observed with lower doses. Below, we report the developmental effects of neonatal administration of OT or antagonist on central patterns of ERα and OT neurons as observed in adult male and female prairie voles.

## Results

**Figure 1:**
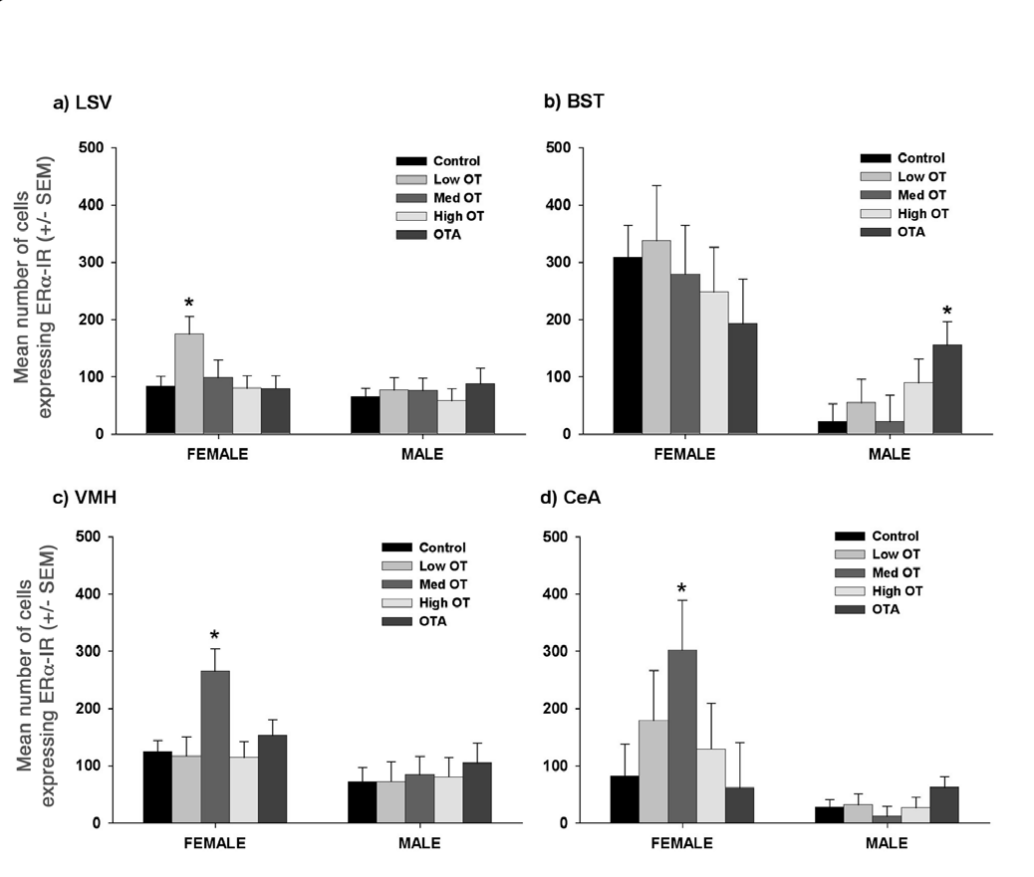
**Developmental effects of oxytocin on central ERα**. The mean number of cells expressing ERα-IR in females and males in (a) the ventral lateral septum (LSV); (b), bed nucleus of the stria terminalis (BST); (c) ventromedial nucleus of the hypothalamus (VMH); and (d), the central amygdala (CeA). The effect of neonatal manipulation of OT was sexually dimorphic, dose-dependent, and site-specific. In females, treatment with OT increased ERα-IR in the LSV (a), VMH (c) and CeA (d). In males, OTA increased ERα-IR in the BST (c). Low, Med, and High treatments were single injections of 0.03 μg OT, 0.3 μg OT, or 3 μg OT, respectively. * = significantly different from CON.

**Figure 2:**
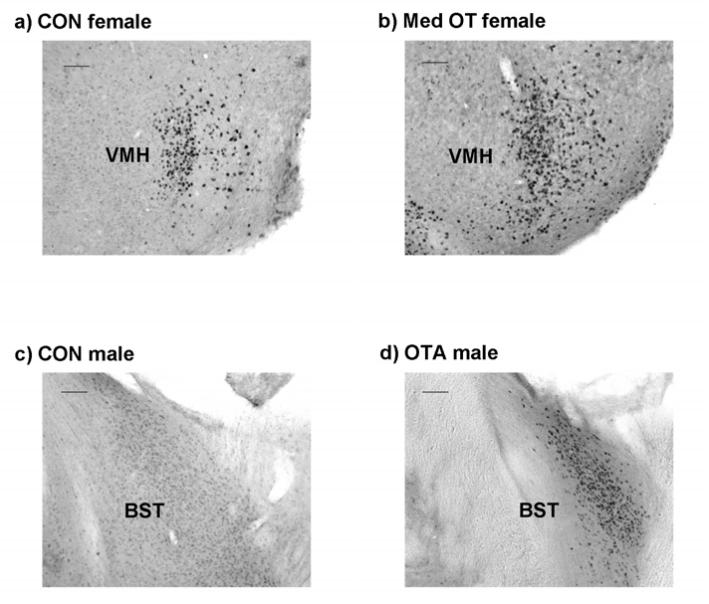
**Central ERα**. Photomicrographs (100x) of ERα-IR in the ventromedial nucleus of the hypothalamus (VMH) of females from the (a) CON and (b) Med OT groups, and in the bed nucleus of the stria terminalis (BST) of males from the (c) CON and (d) OTA groups. The black scale bar represents 200 μm.

**Figure 3:**
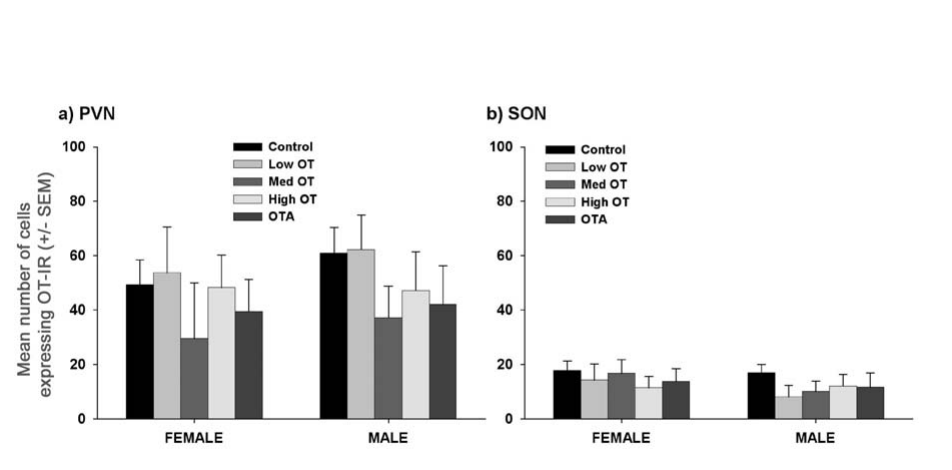
**OT-IR in the hypothalamus**. The mean number of cells expressing OT-IR in females and males in (a) the paraventricular nucleus of the hypothalamus (PVN) and (b) the supraoptic nucleus (SON). There was no significant treatment effect in either nucleus.

Treatment effects were site-specific, sexually dimorphic, and dose-dependent. In males, treatment with OT antagonist (OTA) increased ERα-immunoreactivity (IR) in the bed nucleus of the stria terminalis (BST; *P *< 0.05) (Fig 1 and 2). In females, treatment with Low OT (0.03 μg) resulted in a significant increase in ERα-IR in the ventral lateral septum (LSV), while Med OT (0.3 μg) increased ERα-IR in the ventromedial nucleus of the hypothalamus (VMH; *P *< 0.05) and the central amygdala (CeA; *P *< 0.05) (Fig 1 and 2). There were no apparent treatment effects in the medial preoptic area (MPOA), medial amygdala (MeA), central amygdala (CeA), or arcuate (ARC) in either males or females. However, in males, there was a tendency for OTA to increase ERα-IR in the ARC (*P *< 0.06). In contrast to ERα-IR, there was no treatment effect on the number of OT-IR cells in the PVN or supraoptic nucleus (SON) of the hypothalamus in either males or females (Fig 3).

## Discussion

Previous studies have demonstrated that neonatal manipulation of OT affects the central production of OT [2] and the number and distribution of ERα-IR neurons [3] during the neonatal period, at least up to 21 days after birth. Other studies have shown that neonatal manipulation of OT can have long-term behavioral effects that are expressed a week to months after the initial treatment such as changes in response to social stimuli or reductions in parental care [4,8,9,12]. Taken together these studies predict that the early effects of OT on the organization of the CNS are long-term, and should not only be apparent in neonates, but also in adults. This is supported by a recent study that found alterations in OT content of the pituitary of adult rats resulting from neonatal OT and OTA [10]. The results from our study further support the prediction that neonatal manipulation of OT can alter the adult CNS, as neonatal manipulation of OT on the day of birth affected ERα-IR in both males and females. The results from this study extend the previous findings indicating that the effects of increased or decreased OT during the neonatal period on the CNS are long term. The organizational effects of OT vary with age and are dose-dependent and sexually dimorphic. The results also suggest that interactions between OT and ERα during development are important in the regulation of behavior later in life.

### Developmental effects of OT vary with age and dose

Previous studies, using c-Fos as an indicator of neural activation, demonstrated that neonatal manipulation of OT has immediate effects on the CNS [1]. It is unclear whether the immediate and developmental effects of neonatal OT or OTA result from direct effects on the CNS or result from indirect effects, perhaps through an afferent feedback response to OT stimulation of peripheral organs. If the differential neural activation seen in response to OT or OTA treatment results from direct effects of OT on the brain, there is good reason to expect it to have developmental effects. There is ample opportunity for OT to affect the organization of the CNS as OT receptor (OTR) distributions are still developing during the early postnatal period [13]. The opportunity for OT to alter the CNS also is greater than that predicted by the adult distribution of OTR as some regions such as the cingulate cortex and the medial mammilary nucleus express OTR during the neonatal period but not during adulthood [14]. Recent work has demonstrated that developmental effects of neonatal OT are evident as late as day 21 [2,3]. Neonatal treatment with either High OT (3 μg) or OTA increased the number of OT-IR cells in the PVN of females at day 21 [2], however the present results indicate these effects do not last into adulthood. This is somewhat surprising given that in rats, the same neonatal manipulations alter OT content of the pituitary in adulthood [10], however the regulation of the oxytocinergic system is expected to differ between species showing a high degree of social interactions outside of copulation and species with more limited social interactions such as the rat. Additionally, pituitary OT content and number of OT neurons in the PVN could be differentially regulated. It is possible that alterations in OT-IR were not observed in adult prairie voles because some aspect of physiological and neural maturation from weanling to adult reduces any differences in OT production. Alternatively, differences in OT production might be lost as a result of the changing social environment, from a nest with contact with parents to housing with a single sibling. Finally, an increased sample size could reveal treatment effects but, if so, would suggest the effects are not as robust as those seen at day 21.

OT does, however, have lasting developmental effects on the distribution and number of ERα-IR neurons. Both neonatal OT and OTA altered ERα-IR in adult females, however the regions affected differed from those reported in 21-day old females. In day 21 females, High OT increased ERα-IR in the VMH and OTA decreased ERα-IR in the MPOA [3] but only lower doses, Low OT and Med OT, had effects on ERα-IR lasting into adulthood. The lower doses moved ERα-IR in the same direction as those observed by Yamamoto et al. [3], with both increasing ERα-IR, however the LSV and VMH were affected, not the MPOA. In males, effects also varied depending upon age. Neonatal treatment with OTA had no apparent effects in males at day 21, but by adulthood increases in ERα were evident in the BST.

It is not surprising that the observable effects of neonatal manipulation of OT vary with age. Development of neuroendocrine systems, both in terms of production of neuropeptides and sensitivity to hormones, can occur over a relatively long period of time [e.g., [15]], thus the organizational effects hormones on various brain regions or behaviors might be apparent at different time points. For example, it is clear that in prairie voles, the distribution of ERα continues to change during the second and third postnatal weeks [3] and changes may occur beyond that point. Additionally, some sexual maturation occurs between day 21 and day 60–90, the two ages for which data are available. It is expected that during intermediate ages there are some additional changes in the neuroendocrine system and the developmental effects of OT might be manifested differently after sexual maturation. Dose-dependent effects of OT also were expected given that this is common for many biologically active chemicals and hormones. In order to put the dose-dependent effects observed here into context, future studies measuring endogenous levels of OT and natural variation in central OT will be necessary.

### Sexually dimorphic effects of neonatal OT/OTA

Sexually dimorphic responses to neonatal OT/OTA is consistent with previous studies of the developmental effects of OT on both neuroanatomy [2,3,10] and behavior [8,9,16]. Most studies reporting data for both sexes have found females to be more sensitive than males to the developmental effects of OT on neuroanatomy and behavior [2,3,9,12,16]. Sexually dimorphic effects on ERα-IR, in particular, were expected based on sexual dimorphism in ERα evident in adult prairie voles [17,18] and on differences in ontogeny of the ERα distribution in males versus females [3,19,20]. In the present study, developmental effects of OT were observed in both sexes but the regions and the direction of effect were different for males and for females. In males, OTA increased ERα-IR while in females, OT increased ERα-IR. In light of the regions affected, the direction of response fits with behavioral traits associated with manipulation of OT. OTA increased ERα-IR in the BST of males. Behavioral data suggests that OTA treatment might generally increase anxiety or aggression [8,9] and it has been hypothesized that an increase in ERα in the BST is correlated with increased aggression [21].

### OT regulation of steroid sensitivity

In many species, the effects of OT are steroid-dependent and estrogen increases sensitivity to OT [22-25]. However, in highly social species, such as the prairie vole, it appears that OT regulates sensitivity to steroids. Such an interaction is in keeping with the events leading up to copulation; prairie vole females require a prolonged period of social contact with a male before becoming sexually receptive [26]. Social contact is regulated by OT [27] while E regulates sexual receptivity, thus in a species requiring social contact prior to copulation, OT affects sensitivity to E [28]. Additionally, evidence for OT regulation of E sensitivity come from studies of human breast cancer cell lines in which OT down regulates both ERα mRNA and ERα protein expression [29]. Furthermore, variations in maternal care that are associated with increased OTR also increase ERα expression in the MPOA [30,31]. It is becoming increasingly clear that neuropeptides interact with steroids to regulate behavior (reviewed in [21]) and the results of this study indicate one more way in which neuropeptides might alter sensitivity to steroids. The fact that manipulations of OT early in life alter ERα-IR in adulthood, suggests that OT might not simply regulate sensitivity to E through activational effects, but that organizational effects of OT produce lasting changes in sensitivity to steroids. Our data provide an additional mechanism by which neuropeptides might act to alter or regulate sensitivity to steroids. This in turn has the potential to alter sociosexual behaviors mediated by OT and ERα.

## Conclusion

The results from this study advance findings from the previous studies that demonstrated organizational effects of OT during the neonatal period on ERα and OT expression. First, the effects on ERα expression/organization are long-term, being expressed in adulthood. Second, the effects were not only sexually dimorphic, but the observed effect changed with age. Significant changes in ERα expression in males occurred at some point between weaning and adulthood while in females, significant changes were observed at both ages. In contrast to the observed organizational effects on ERα, the effect on OT neurons in the PVN reported in juveniles were no longer observed in adults, suggesting that changes to the oxytocinergic system are temporary. Third, alterations in ERα-IR were site-specific and likely to be behaviourally relevant. For example, antagonist increased ERα-IR in the BST and high levels of ERα are associated with decreases in social behavior, as is blockade of OTR early in life. There is mounting evidence for the role of OT in the development of the CNS, perhaps mediating social behavior later in life. The data reported here provide further support for this hypothesis. More important, these data demonstrate that a neuropeptide can act during developmental periods to affect sensitivity to steroids later in life and indicates the importance of considering both neuropeptides and steroids in examining developmental effects of hormones on subsequent social behavior and underlying neuroanatomy.

## Methods

### Husbandry and Treatments

Animals used in this study were laboratory-reared prairie voles that originated from wild stock trapped near Urbana, Illinois. Animals were housed under a 14:10 light:dark cycle and provided high fiber rabbit chow and water ad libitum. On the day of birth pups were sexed and marked for identification. Pups were given a single intraperitoneal injection (50 μl) of isotonic saline (SAL; vehicle control), one of three doses of OT, 3 μg (approximately 1 μg/g; High OT), 0.3 μg (approximately 0.1 μg/g; Med OT), 0.03 μg (approximately 0.01 μg/g; Low OT), or 0.3 μg (approximately 0.1 μg/g) OT antagonist (OTA; [d(CH_2_)_5_, Tyr(Me)^2^, Orn^8^]-Vasotocin; Bachem, Belmont, CA). An additional group was handled but not injected (HAN). The doses for High OT and for OTA were chosen because there is extensive literature indicating that during the neonatal period these doses can affect the CNS immediately after injection [1] and have developmental effects on a variety of physiological and behavioral responses in both male and female rats and prairie voles [2,4-9]. A relatively low dose of OTA was chosen because the antagonist binds OTR more effectively than OT, with an affinity for OTR greater than 10 times that of the natural ligand [32] and this same dose has been used in multiple other studies [2,3,7,9]. Injections were intraperitoneal because of the disruptive nature of central injections. Previous work demonstrated that the OTA dose and High OT cause differential c-Fos-IR in neonates, indicating that an i.p. route does affect the CNS [1]. However, it is unknown whether the immediate effects of these treatments are direct or indirect. It is possible that OT and/or OTA cross the blood-brain barrier as it is not yet fully developed in the first 2 postnatal weeks [33] and some data indicate that small neuropeptides can cross the blood-brain barrier [34].

Pups were assigned to treatment groups randomly with the restriction that within each litter there was at least one control (SAL or HAN) and one experimental (OT or OTA) pup. No treatment was represented more than once per sex per litter. Pups were kept warm during the injections using a heat lamp and returned as a group to the home cage within 10 min. Pups were weaned at 21 days of age and then housed in same-sex sibling pairs until brains were collected (see below) between 60 and 90 days of age; *n *= 6 per treatment per sex. Animals were housed in accordance with the USDA and NIH guidelines and measures were taken to minimize pain and discomfort. All procedures were approved by the University of Illinois at Chicago Animal Care and Use Committee.

### Tissue collection and labelling of ERα and OT neurons

The following is a brief description of tissue collection and immunocytochemistry; for complete details see [17] for ERα and [2] for OT. All tissue was collected between 3–5 hr after lights on. Animals were deeply anaesthetized using a combination of Ketamine and Xylazine and then decapitated. Brains were removed and fixed using a spinning immersion technique [35] with 4% paraformaldehyde and 5% acrolein in 0.1 M KPBS (pH 7.6). After 4 hr in fixative (fix was refreshed at 2 hr) brains were removed and stored in 25% sucrose at 4°C until sectioning at 30 μm on a freezing sliding microtome. Free-floating sections were then stored in cryoprotectant [36] until processed using standard ABC (avidin:biotinylated enzyme complex) immunocytochemistry.

Sections were rinsed in 0.05M KPBS and then incubated at room temperature in 1% sodium borohydride. Sections were again rinsed in KPBS and then incubated in primary antibody, rabbit ERα polyclonal antibody (Upstate USA, Charlottesville, VA anti-ERα C1355) at a dilution of 1:8,000 in 0.05M KPBS-0.4% TritonX-100 or anti-OT (generously provided by Dr. Mariana Morris) at a dilution of 1:100,000 for 1 hr at room temperature and then for 48 hr at 4°C. The ERα antibody binds both free and bound receptors, minimizing variation due to endogenous steroid levels [37]. The antibody was generated against the last 15 C-terminal amino acids of the rat ERα protein, a region that shares no homology with ERβ. The specificity of this antibody was tested by omitting the primary antibody from the ICC procedure and by performing ICC after pre-adsorption with the synthetic peptide (10x the concentration of the antibody). Tests of specificity are also detailed in Cushing and Wynne-Edwards [17]. Following incubation in the primary, tissue was rinsed and then incubated for 1 hr at room temperature in biotinylated goat, anti-rabbit IgG (1:600 dilution in KPBS-0.4% TritonX-100; Vector Laboratories, Burlingame, CA). Sections were again rinsed prior to being incubated for 1 hr at room temperature in an avidin-biotin peroxidase complex (Vectastain ABC kit-elite pk-6100 standard, 4.5 μl A and 4.5 μl B per 1 ml solution; Vector Laboratories) diluted in KPBS-0.4% TritonX-100. Sections were rinsed with KPBS followed with rinses using 0.175M sodium acetate. Finally, ERα or OT was visualized by incubating in a nickel sulfate-diaminobenzidine chromogen (ERα) or a diaminobenzidine (OT) solution for 15 min. Sections were rinsed and then mounted on subbed glass slides, air dried, dehydrated in ascending ethanol solutions, cleared in Histoclear, and coverslipped using Histomount.

### Statistical Analysis

For each brain region, the number of cells expressing ERα- or OT-immunoreactivity (IR) were counted bilaterally using IP Lab (Scanalytics, Inc, Fairfax, VA) image analysis software at 40× magnification and then averaged. Slides were coded and then scored by an observer who was blind to the treatment condition of the subject. Regions analyzed for ERα-IR included the ventral lateral septum (LSV), medial preoptic area (MPOA), bed nucleus of the stria terminalis (BST), arcuate nucleus (ARC), ventromedial nucleus of the hypothalamus (VMH), the central amygdala (CeA), and the medial amygdala (MeA). Efforts were made to score similar sections of each region for all subjects by using the shape of the nucleus, landmarks such as the optic tracts, and referencing the mouse and rat brain atlases [38,39]. These areas were chosen because they are involved in regulating a variety of aspects of reproduction and social behavior, behaviors that have been reported to respond to neonatal manipulation of OT [7,4,8,16]. OT-IR cells were counted in the paraventricular nucleus of the hypothalamus (PVN) and in the supraoptic nucleus (SON), which are the two primary brain regions that produce OT.

Because there was no difference between the vehicle control (SAL) and the handling control (HAN) in ERα-IR in either sex or any region, for the purpose of analysis these were grouped into a single control group (CON). Although multiple doses of OT were used, the *apriori *goal of this study was to determine the effect of treatment relative to controls and not to generate a dose response curve. Therefore, treatment differences were compared against the control group using a Fisher's Least Significant Difference (LSD) test for each sex by region. A Fisher's LSD was used because it is the recommended statistical approach for *apriori *comparisons [40]. Sexes were analyzed separately because *apriori *we expected there to be significant differences between males and females based on previously published studies of developmental effects of OT [2,3,8,9,16]. Results were considered significant if *P *< 0.05.

## Authors' contributions

KMK carried out neonatal manipulations and tissue collection, oversaw work of SY and EP, and wrote the manuscript. SY carried out neonatal manipulations, tissue collection, and slicing. EP stained the tissue for ERα and OT. BSC designed the study, collected neuroanatomical data, and edited the manuscript. All authors have read and approved the submitted manuscript.

## References

[B1] Cushing BS, Yamamoto Y, Carter CS, Hoffman GE (2003). Central c-Fos expression in neonatal male and female prairie voles in response to treatment with oxytocin. Dev Brain Res.

[B2] Yamamoto Y, Cushing BS, Kramer KM, Epperson P, Hoffman GE, Carter CS (2004). Neonatal manipulations of oxytocin alter expression of oxytocin and vasopressin immunoreactive cells in the paraventricular nucleus of the hypothalamus in a gender specific manner. Neuroscience.

[B3] Yamamoto Y, Carter CS, Cushing BS (2006). Neonatal manipulation of oxytocin affects expression of estrogen receptor alpha. Neuroscience.

[B4] Cushing BS, Levine K, Cushing NL (2005). Neonatal manipulations of oxytocin affect reproductive behavior and reproductive success of adult female prairie voles (*Microtus ochrogaster*). Horm Behav.

[B5] Sohlström A, Olausson H, Brismar K, Uvnäs-Moberg K (2002). Oxytocin treatment during early life influences reproductive performance in ad libitum fed and food-restricted female rats. Biol Neonate.

[B6] Uvnäs-Moberg K, Alster P, Petersson M, Sohlström A, Bjorkstrand E (1998). Postnatal oxytocin injections cause sustained weight gain and increased nociceptive thresholds in male and female rats. Ped Res.

[B7] Withuhn TF, Kramer KM, Cushing BS (2003). Early exposure to oxytocin affects the age of vaginal opening and first estrus in female rats. Physiol Behav.

[B8] Bales KL, Pfeifer LA, Carter CS (2004). Sex differences and developmental effects of manipulations of oxytocin on alloparenting and anxiety in prairie voles. Dev Psychobiol.

[B9] Kramer KM, Cushing BS, Carter CS (2003). Developmental effects of oxytocin on stress response: single versus repeated exposure. Physiol Behav.

[B10] Young E, Carter CS, Cushing BS, Caldwell JD (2005). Neonatal manipulation of oxytocin alters oxytocin levels in the pituitary of adult rats. Horm Metabol Res.

[B11] Kramer KM, Cushing BS, Carter CS, Wu J, Ottinger MA (2004). Sex and species differences in plasma oxytocin using an enzyme immunoassay. Can J Zool.

[B12] Kramer KM, Choe C, Carter CS, Cushing BS (2006). Developmental effects of oxytocin on neural activation and neuropeptide release in response to social stimuli. Horm Behav.

[B13] Shapiro LR, Insel TR (1989). Ontogeny of oxytocin receptors in rat forebrain: A quantitative study. Synapse.

[B14] Gimpl G, Fahrenholz F (2001). The oxytocin receptor system: structure, function, and regulation. Physiol Rev.

[B15] Johansen JA, Jordan CL, Breedlove SM (2004). Steroid hormone masculinization of neural structure in rats: a tale of two nuclei. Physiol Behav.

[B16] Bales KL, Carter CS (2003). Sex differences and developmental effects of oxytocin on aggression and social behavior in prairie voles (*Microtus ochrogaster*). Horm Behav.

[B17] Cushing BS, Wynne-Edwards KE (2006). Estrogen receptor-α distribution in male rodents is associated with social organization. J Comp Neurol.

[B18] Hnatczuk OC, Lisciotto CA, DonCarlos LL, Carter CS, Morrell JI (1994). Estrogen receptor immunoreactivity in specific brain areas of the prairie vole (*Microtus ochrogaster*) is altered by sexual receptivity and genetic sex. J Neuroendocrinol.

[B19] Kuhnemann S, Brown TJ, Hochberg RB, MacLusky NJ (1994). Sexual differences in the development of estrogen receptors in the rat brain. Horm Behav.

[B20] Yokosuka M, Okamura H, Hayashi S (1997). Postnatal development and sex differences in neurons containing estrogen receptor-alpha immunoreactivity in the preoptic brain, the diencephalon, and the amygdala in the rat. J Comp Neurol.

[B21] Cushing BS, Kramer KM (2005). Mechanisms underlying epigenetic effects of early social experience: the role of neuropeptides and steroids. Neurosci Biobehav Rev.

[B22] Coirini H, Johnson AE, McEwen BS (1989). Estradiol modulation of oxytocin binding in the ventromedial hypothalamic nucleus of male and female rats. Neuroendocrinology.

[B23] Johnson AE (1992). The regulation of oxytocin receptor binding in the ventromedial hypothalamic nucleus by gonadal steroids. Ann NY Acad Sci.

[B24] McCarthy MM, Kleopoulos SP, Mobbs CV, Pfaff DW (1994). Infusion of antisense oligodeoxynucleotides to the oxytocin receptor in the ventromedial hypothalamus reduces estrogen-induced sexual receptivity and oxytocin receptor-binding in the female rat. Neuroendocrinology.

[B25] Witt DM (1997). Regulatory mechanisms of oxytocin-mediated sociosexual behavior. Ann NY Acad Sci.

[B26] Carter CS, Witt DM, Schneider J, Harris ZL, Volkening D (1987). Male stimuli are necessary for female sexual behavior and uterine growth in prairie voles (*Microtus ochrogaster*). Horm Behav.

[B27] Carter CS, DeVries AC, Getz LL (1995). Physiological substrates of mammalian monogamy – the prairie vole model. Neurosci Biobehav Rev.

[B28] Cushing BS, Carter CS (1999). Prior exposure to oxytocin mimics social contact and facilitates sexual behaviour in females. J Neuroendocrinol.

[B29] Cassoni P, Catalano MG, Sapino A, Marrocco T, Fazzari A, Bussolati G, Fortunati N (2002). Oxytocin modulates estrogen receptor alpha expression and function in MCF7 human breast cancer cells. Inter J Oncol.

[B30] Champagne FA, Weaver ICG, Diorio J, Sharma S, Meaney MJ (2003). Natural variations in maternal care are associated with estrogen receptor α expression and estrogen sensitivity in the medial preoptic area. Endocrinology.

[B31] Francis DD, Young LJ, Meaney MJ, Insel TR (2002). Naturally occurring differences in maternal care are associated with the expression of oxytocin and vasopressin (V1a) receptors: gender differences. J Neuroendocrinol.

[B32] Barberis C, Tribollet E (1996). Vasopressin and oxytocin receptors in the central nervous system. Crit Rev Neurobiol.

[B33] Johanson CE (1980). Permeability and vascularity of the developing brain: cerebellum vs cerebral cortex. Brain Res.

[B34] Banks WA, Kastin AJ (1985). Permeability of the blood-brain barrier to neuropeptides: the case for penetration. Psychoneuroendocrinology.

[B35] Cushing BS, Klein D, Hoffman GE, Carter CS, Le WW, De Vries GJ (2001). Comparison of fixation techniques: immersion versus perfusion. Horm Behav.

[B36] Watson RE, Wiegand SJ, Clough RW, Hoffman GE (1986). Use of cryoprotectant to maintain long-term peptide immunoreactivity and tissue morphology. Peptides.

[B37] Murphy AZ, Shupnik MA, Hoffman GE (1999). Androgen and estrogen (α) receptor distribution in the periaqueductal gray of the male rat. Horm Behav.

[B38] Paxinos G, Watson C (1998). The Rat Brain in Stereotaxic Coordinates.

[B39] Paxinos G, Franklin KBJ (2001). The Mouse Brain in Stereotaxic Coordinates.

[B40] Sokal RR, Rohlf FJ (1995). Biometry: the principles and practice of statistics in biological research.

